# Social Inequalities in Infant Mortality Related to Congenital Anomalies: A Population‐Based Register Study

**DOI:** 10.1111/ppe.70162

**Published:** 2026-06-01

**Authors:** Bermet Amirova, Nathalie Lelong, Marine Wojciechowski, Marie‐Victoire Sénat, Babak Khoshnood, Jennifer Zeitlin, Isabelle Monier

**Affiliations:** ^1^ Université Paris Cité and Université Sorbonne Paris Nord, INSERM, INRAE, Centre for Research in Epidemiology and Statistics Paris France; ^2^ Clinique Des Femmes Paris France

**Keywords:** antenatal detection, congenital anomalies, deprivation index, infant mortality, social inequalities

## Abstract

**Background:**

Infant mortality has recently increased in France, especially in deprived areas. Congenital anomalies (CA) are a major cause of infant mortality and could contribute to these trends.

**Objectives:**

This study aimed to investigate socio‐spatial inequalities in infant mortality associated with CA. It also examined socio‐spatial differences in antenatal detection.

**Methods:**

We included all live births ≥ 22 weeks with CA from the Paris Registry of CA (remaPAR). Births are linked to infant deaths < 1 year using vital statistics records. A case‐population design was applied to assess the association between an area‐based deprivation score, assigned at the smallest census tract level (IRIS) of the mother's place of residence, and risks of infant death and live birth with CA. In the absence of data on births at the IRIS level, we used an aggregated population comparison group of children < 2 years old in 2019–2022 census data. We computed unadjusted odds ratios (OR) by comparing the distribution of the area‐based score in quartiles in the case and census comparison populations. We also compared antenatal detection of CA by deprivation quartile.

**Results:**

The study included 2539 live births, resulting in 81 infant deaths. The proportions of infant deaths with CA were 39.5% and 16.0% in the most and least deprived quartiles, respectively, yielding an OR of 2.43 (95% confidence interval [CI] 1.28, 4.63). For live births with CA, these percentages were 29.7% and 22.0% with an OR of 1.34 (95% CI 1.20, 1.49). No variation across quartiles was observed in antenatal detection of CA, with a detection rate of 56.0% for live births and 85.2% for infant deaths. First‐trimester ultrasound use was high (90.5%) but was lower in the most compared to the least deprived quartile (87.8% and 93.9%, respectively); however, second‐trimester ultrasound use was similar across quartiles.

**Conclusions:**

Area‐based deprivation was associated with higher risks of infant mortality and live birth with CA, but not antenatal detection of CA. These results raise questions about differences in CA aetiology and decision‐making about termination of pregnancy after CA detection.

## 1 Background

The infant mortality rate (IMR, death < 365 days of life) is a key indicator of a country's health system performance and perinatal health. The IMR has decreased dramatically in the last century, but recent data shows that rates are stagnating or increasing in some countries [1]. In France, the IMR increased from 3.5 per 1000 live births in 2011 to 4.0 per 1000 live births in 2023 [2], mainly driven by neonatal deaths (< 28 days). Growing evidence links these trends to social and health inequalities, with higher neonatal mortality in deprived urban areas [3, 4].

Congenital anomalies (CA) affect approximately 3%–4% of all births [5] and are one of the leading causes of infant mortality, contributing to between 20% and 30% of deaths [6] and to long‐term morbidity and disability [7]. Recent studies from high‐income countries show that socioeconomic status (SES) is associated with CA prevalence, access to prenatal screening and decisions to terminate a pregnancy following antenatal detection of CA [8, 9, 10, 11]. While France is an interesting setting to explore inequalities in CA‐related mortality, given universal maternity care and high uptake of routine trimester ultrasounds [12], data limits have restricted research. French CA registers include some information on maternal SES, but they do not collect data in control populations and comprehensive population birth sources describing maternal SES are not available in France.

Using new data on area‐based deprivation from the Parisian CA register, the objective of the study was to investigate socio‐spatial inequalities in infant deaths and live births with CA. We also examined socio‐spatial differences in antenatal detection of CA.

## 2 Methods

### 2.1 Study Design

We used a case‐population study design [13], which compares exposure in a case population to a measure of exposure derived from aggregated population data. It is applied when control populations are not available and is suitable for rare outcomes [13, 14, 15, 16].

### 2.2 Cases

Data came from the Paris Registry of congenital anomalies (remaPAR) which records all births with CAs detected during pregnancy and the early neonatal period, among women residing and delivering in Paris [7]. Case ascertainment of major anomalies, in line with EUROCAT guidelines [17], relies on active surveillance, using multiple data sources, including medical records in maternity, neonatology and foetopathology departments. Mortality is ascertained until 1 year of age based on hospital records and vital statistics. Since 2019, maternal addresses at the time of conception have been collected for geocoding at the smallest census tract zone (IRIS) [18]. The study population included all live births with CA in 2019–2023 (*N* = 2577).

### 2.3 Comparison Population

The comparison group was derived from population census data. Because birth data were unavailable at the IRIS level, we used counts of children aged < 2 years from 2019 to 2022 to approximate the spatial distribution of the underlying population at risk. Census data for 2023 were not yet available. CA cases could not be excluded from the comparison population.

### 2.4 Exposure

The exposure was the area‐based deprivation score of the mother's place of residence at the IRIS level, which includes about ~2000 residents (*N* = 992 in Paris). Deprivation was assessed using the Perinatal French Deprivation Index (P‐FDep) which is a composite measure derived through principal component analysis of sociodemographic indicators (median household income, unemployment rate and proportions of immigrants, non‐homeowners and single‐parent families) [4]. The census reference population was used to derive quartiles of deprivation. We categorised the score by quartiles, as opposed to quintiles or more granular groups, to ensure an adequate number of cases of infant deaths per group.

### 2.5 Outcomes

The outcomes were infant deaths and live births with CA. A secondary outcome was the antenatal detection of CA, defined as the detection of at least one CA during pregnancy.

### 2.6 Statistical Analysis

Unadjusted odds ratios (OR) were calculated by comparing quartiles of the area‐based deprivation score between cases (infant deaths and live births with CA) and the comparison population at the IRIS level. The least deprived quartile (Q1) was used as a reference.

Additional analyses among CA cases examined antenatal detection status, use of routine ultrasounds and perinatal characteristics (gestational age at birth, timing of death and CA subgroups) by deprivation quartile [19].

### 2.7 Missing Data

We excluded cases with incomplete or erroneous addresses (*n* = 38; 1%). For IRIS units with missing census variables (1.3%), missing values were replaced with the mean of contiguous units.

### 2.8 Sensitivity Analyses

Using vital statistics data on births which are available at the district (*arrondissement*) level, we compared the OR for live births and infant deaths using births and children < 2 year as population comparison groups. This sensitivity analysis was conducted to assess whether children < 2 year provide a valid approximation of the birth population. We modelled the same deprivation score, available at the district level for 2021, in quintiles to account for data skewness that arose when using quartiles due to a small number of arrondissements (*N* = 20).

All analyses were performed in R version 4.4.2 using RStudio.

### 2.9 Ethics Statement

Paris Registry of congenital anomalies has ethics approval from the national data protection authority (Commission Nationale de l'Informatique et des Libertés [no. DR‐2016‐393]).

## 3 Results

There were 2539 live births resulting in 81 infant deaths with CA. Both outcomes were more frequent in the most deprived (29.7% and 39.5%, respectively) than the least deprived quartile (22.0% and 16.0%), based on quartiles derived from the comparison population (*N* = 230,971) (Table 1). The corresponding ORs were 1.34 (95% CI 1.20, 1.49) and 2.43 (95% CI 1.28, 4.63).

**TABLE 1:** Live births and infant deaths with congenital anomalies according to area‐based social deprivation quartiles.

P‐FDep	Control population	Live birth (*n* = 2530)		Infant deaths (*n* = 81)	
	*n* (%)	*n* (%)	OR (95% CI)	*n* (%)	OR (95% CI)
Q1 (least deprived)	57,469 (24.9)	558 (22.0)	1.00 (Reference)	13 (16.0)	1.00 (Reference)
Q2	58,967 (25.5)	576 (22.7)	1.00 (0.89–1.13)	16 (19.8)	1.20 (0.58–2.49)
Q3	56,338 (24.4)	650 (25.6)	1.19 (1.06–1.33)	20 (24.7)	1.57 (0.78–3.16)
Q4 (most deprived)	58,197 (25.2)	755 (29.7)	1.34 (1.20–1.49)	32 (39.5)	2.43 (1.28–4.63)

*Note:* (1) P‐FDep: an area‐based deprivation score derived from the unemployment rate, mean income and proportions of non‐home ownership, single parent households and migrants from 2019 census data. (2) Control population is derived from 2019 to 2022 census data on children < 2 years residing in Paris (census data for 2023 is not yet available).

The CA was detected in 56.0% of live births and 85.2% of infant deaths with CA. These proportions did not vary across quartiles (Table 2). First‐trimester ultrasound coverage was high (90.5%) but was lower in deprived areas (87.8% in Q4 vs. 93.9% in Q1); while second‐trimester ultrasound use was the same across quartiles.

**TABLE 2:** Antenatal detection of congenital anomalies among live births and infant deaths by area‐based social deprivation quartiles.

P‐FDep	Overall (*n* = 2539)	Q1 (*n* = 558)	Q2 (*n* = 576)	Q3 (*n* = 650)	Q4 (*n* = 755)
	*n* (%)	*n* (%)	*n* (%)	*n* (%)	*n* (%)
Live births with congenital anomalies					
1st trimester ultrasound	2259 (90.5)	508 (93.9)	533 (93.7)	562 (88.1)	656 (87.8)
2nd trimester ultrasound	2459 (97.7)	539 (98.5)	557 (97.2)	625 (96.7)	738 (98.1)
Antenatal detection any	1417 (56.0)	325 (58.6)	326 (56.9)	355 (54.7)	411 (54.7)
Infant deaths with congenital anomalies	81 (3.2)	13 (2.3)	16 (2.8)	20 (3.1)	32 (4.2)
1st trimester ultrasound	64 (82.1)	8 (66.7)	12 (80.0)	17 (89.5)	27 (84.4)
2nd trimester ultrasound	79 (98.8)	12 (100)	15 (93.8)	20 (100)	32 (100)
Antenatal detection any	69 (85.2)	11 (84.6)	14 (87.5)	17 (85.0)	27 (84.4)

*Note:* P‐FDep: an area‐based deprivation score derived from the unemployment rate, mean income and proportions of non‐home ownership, single parent households and migrants from 2019 census data. Q1 is the least deprived quartile, Q4 is the most deprived quartile.

Table 3 describes the characteristics of infant deaths associated with CA, showing that 46.9% occurred in the first week and 59.3% took place after term live birth.

**TABLE 3:** Characteristics of infant deaths by area‐based social deprivation quartiles.

P‐FDep	Overall (*n* = 81)	Q1 (*n* = 13)	Q2 (*n* = 16)	Q3 (*n* = 20)	Q4 (*n* = 32)
	*n* (%)	*n* (%)	*n* (%)	*n* (%)	*n* (%)
Gestational age (weeks)					
< 37	33 (40.7)	7 (53.8)	6 (37.5)	< 5	16 (50.0)
≥ 37	48 (59.3)	6 (46.2)	10 (62.5)	16 (80.0)	16 (50.0)
Timing of death					
< 7	38 (46.9)	< 5	9 (56.3)	6 (30.0)	19 (59.4)
7–28	19 (23.5)	6 (46.2)	< 5	6 (30.0)	< 5
28+	24 (29.6)	< 5	< 5	8 (40.0)	9 (28.1)
Congenital anomalies subgroup					
Genetic (including chromosomal)	34 (42.0)	5 (38.5)	6 (37.5)	8 (40.0)	15 (46.9)
Non‐genetic anomalies	47 (58.0)	8 (61.5)	10 (62.5)	12 (60.0)	17 (53.1)

*Note:* P‐FDep: an area‐based deprivation score derived from the unemployment rate, mean income and proportions of non‐home ownership, single parent households and migrants from 2019 census data. Q1 is the least deprived quartile, Q4 is the most deprived quartile. Counts under 5 are suppressed.

In sensitivity analyses, the classification of districts into quintiles was the same when using births or children < 2 years old as the comparison population (Table S1). The ORs derived from both populations showed similar gradients across deprivation quintiles.

## 4 Comment

### 4.1 Principal Findings

Area‐based socioeconomic deprivation was associated with a higher risk of infant death and live birth with CA. Antenatal detection of CA for live births and infant deaths was similar across quartiles. Among live births with CA, first‐trimester ultrasound use was lower in the most (87.8%) compared to the least deprived (93.9%) areas.

### 4.2 Strengths of the Study

The study's strengths are the high‐quality, comprehensive data on CA from a population‐based registry. The high spatial resolution of geocoded maternal addresses at the IRIS level goes beyond previous research on SES inequalities in infant mortality in France, based only on the district [4], and allows estimation of SES inequalities in CA that were previously unavailable.

### 4.3 Limitations of the Data

The absence of individual‐level data to construct a control population led us to adopt a case‐population design using census counts of children < 2 years to estimate the distribution of the exposure in the general population. Reassuringly, sensitivity analyses at the district level showed similar results when using births as a comparison population. Another limitation of this design is the inability to adjust for individual‐level characteristics. Finally, data on the principal cause of death were unavailable, but we were able to describe the type of CA and the timing of death.

### 4.4 Interpretation

This study found that infant death and live births with CA were higher in deprived areas, in concordance with findings from other high‐income countries [6, 9, 11, 20, 21]. Recent spatiotemporal analyses in France have revealed increases in infant mortality in deprived areas with larger migrant populations [3, 4]. Since CA is a major contributor to infant deaths [22], these socio‐spatial disparities may contribute to France's recent rise in neonatal mortality [5, 6, 23].

Nearly half of infant deaths occurred within the first week, with the majority in the most deprived quartile, suggesting that severe CA, which are eligible for termination of pregnancy for foetal anomaly, drive these early deaths. These disparities in mortality could be related to differential access and quality of care and/or differences in parental decision‐making after detection which may vary by SES and geographical origin [11, 24], warranting further investigation. Although overall antenatal detection rates did not vary by area‐level deprivation, a reassuring finding in a country with universal maternity coverage, the uptake of first trimester ultrasound was lower in more deprived areas, indicating suboptimal healthcare use, possibly due to differences in screening access, timing or utilisation by SES [25].

These differences could also be related to CA characteristics or other pregnancy complications associated with the CA. The higher number of live births with CA in more deprived areas may reflect a greater exposure to established risk factors, including maternal diabetes or obesity, health behaviours (smoking, folic acid supplementation), and consanguinity [9, 26, 27].

While this study was motivated by the investigation of infant mortality, the higher proportion of live births with CA in deprived areas raises questions about the psychosocial impact and economic burden of long‐term care for children with CA on already disadvantaged families with implications for health service planning [28, 29, 30].

## 5 Conclusions

Area‐based socioeconomic deprivation was associated with higher risks of infant death and live birth with CA, while overall antenatal detection rates were similar across deprivation levels. These results underscore the importance of future research to investigate post‐detection parental decisions, the aetiology and health complications associated with CA, and the health service and other needs of surviving children with CA.

## Author Contributions

I.M. and J.Z. designed the study. B.A. conducted the formal analysis. B.A. wrote the first draft of the manuscript. All authors critically reviewed the manuscript and approved the final version of the manuscript.

## Funding

The study was funded by ARS (Agence Régionale de Santé) Ile‐de‐France. The registry, remaPAR receives structural funding from INSERM and Santé publique France.

## Conflicts of Interest

The authors declare no conflicts of interest.

## Supporting information


**Table S1:** Risk of live birth and infant death with congenital anomalies by quantile of socioeconomic deprivation using births versus children < 2 years of age as control populations at the district‐level in Paris.

## Data Availability

The data that support the findings of this study are from the Paris Registry of congenital anomalies (remaPAR). Due to privacy and ethical restrictions related to the use of confidential data, the data used in this study cannot be made publicly available.
