# Supplementary material for: Social Inequalities in Infant Mortality Related to Congenital Anomalies: A Population‐Based Register Study
Source: Paediatr Perinat Epidemiol. 2026 Jun 1;40(6):694–9. doi: 10.1111/ppe.70162 (PMC13522641; doi:10.1111/ppe.70162)
Supplement: Supplementary file 1 [file PPE-40-694-s001.docx]

**Supplementary Tables**

Supplementary Table 1. Risk of live birth and infant death with congenital anomalies by quantile of socioeconomic deprivation using births versus children <2 years of age as control populations at the district-level in Paris

| P-FDep | Births  N=25810 | < 2 years N=58421 | Live births N=2,539 | | | Infant deaths N=81 | | |
| --- | --- | --- | --- | --- | --- | --- | --- | --- |
|  | n (%) | n (%) | n (%) | OR*  (95% CI) | OR**  (95% CI) | n (%) | OR*  (95% CI) | OR**  (95% CI) |
| Q1 | 7253 (28.1) | 16124  (27.6) | 530 (13.9) | 1.00 (Reference) | 1.00 (Reference) | 15 (18.5) | 1.00 (Reference) | 1.00 (Reference) |
| Q2 | 4655 (18.0) | 10572  (18.1) | 463 (12.1) | 1.40  (1.23–1.60) | 1.35  (1.19–1.53) | 12 (14.8) | 1.25  (0.58–2.67) | 1.22  (0.57–2.61) |
| Q3 | 4677 (18.1) | 10121  (17.3) | 475 (12.4) | 1.43  (1.26–1.63) | 1.45  (1.28–1.64) | 15 (18.5) | 1.55  (0.76–3.18) | 1.59  (0.78–3.26) |
| Q4 | 4606 (17.8) | 10285  (17.6) | 492 (12.9) | 1.52  (1.33–1.73) | 1.48  (1.30–1.68) | 17 (21.0) | 1.79  (0.89–3.58) | 1.78  (0.89–3.56) |
| Q5 | 4619 (17.9) | 11319  (19.4) | 579 (15.1) | 1.82  (1.61–2.06) | 1.59  (1.41–1.79) | 22 (27.2) | 2.31  (1.20–4.46) | 2.09  (1.08–4.03) |
| *OR calculated using P-FDep quintiles derived by weighting by births  ** OR calculated using P-FDep quintiles derived by weighting by control population, i.e. children under <2 years old  NOTE: (1) P-FDep: an area-based deprivation score derived from the unemployment rate, mean income and proportions of non-home ownership, single parent households and migrants. (2) control population is derived from 2021 census data on children <2 years residing in Paris. (3) Quintiles were used instead of quartiles to account for data skewness due to a small number of units on the district level (N=20), compared to the IRIS level (N=992). (4) 2021 census data used for P-FDep due to already available scores by district levels in France.  Q1 is the least deprived quintile, Q5 is the most deprived quintile. | | | | | | | | |
